# PRAS40 suppresses atherogenesis through inhibition of mTORC1-dependent pro-inflammatory signaling in endothelial cells

**DOI:** 10.1038/s41598-019-53098-1

**Published:** 2019-11-14

**Authors:** Kevin Sun Zhang, Johannes Schecker, Alexandros Krull, Eva Riechert, Lonny Jürgensen, Verena Kamuf-Schenk, Jana Burghaus, Leon Kiper, Thanh Cao Ho, Kerstin Wöltje, Verena Stangl, Hugo A. Katus, Karl Stangl, Mirko Völkers, Till F. Althoff

**Affiliations:** 1 Department of Cardiology, Angiology, and Pneumology, University Hospital Heidelberg, University of Heidelberg, Heidelberg, Germany; 2 Charité – University Medicine Berlin, Department of Cardiology and Angiology, Charité Campus Mitte, Berlin, Germany; 3 DZHK (German Centre for Cardiovascular Research), partner site Heidelberg/Mannheim, Heidelberg, Germany; 4 DZHK (German Centre for Cardiovascular Research), partner site Berlin, Berlin, Germany

**Keywords:** Cardiovascular biology, Atherosclerosis

## Abstract

Endothelial pro-inflammatory activation plays a pivotal role in atherosclerosis, and many pro-inflammatory and atherogenic signals converge upon mechanistic target of rapamycin (mTOR). Inhibitors of mTOR complex 1 (mTORC1) reduced atherosclerosis in preclinical studies, but side effects including insulin resistance and dyslipidemia limit their clinical use in this context. Therefore, we investigated PRAS40, a cell type-specific endogenous modulator of mTORC1, as alternative target. Indeed, we previously found PRAS40 gene therapy to improve metabolic profile; however, its function in endothelial cells and its role in atherosclerosis remain unknown. Here we show that PRAS40 negatively regulates endothelial mTORC1 and pro-inflammatory signaling. Knockdown of PRAS40 in endothelial cells promoted TNFα-induced mTORC1 signaling, proliferation, upregulation of inflammatory markers and monocyte recruitment. In contrast, PRAS40-overexpression blocked mTORC1 and all measures of pro-inflammatory signaling. These effects were mimicked by pharmacological mTORC1-inhibition with torin1. In an *in vivo* model of atherogenic remodeling, mice with induced endothelium-specific PRAS40 deficiency showed enhanced endothelial pro-inflammatory activation as well as increased neointimal hyperplasia and atherosclerotic lesion formation. These data indicate that PRAS40 suppresses atherosclerosis via inhibition of endothelial mTORC1-mediated pro-inflammatory signaling. In conjunction with its favourable effects on metabolic homeostasis, this renders PRAS40 a potential target for the treatment of atherosclerosis.

## Introduction

Atherosclerosis is an inflammatory disorder of large and medium-sized arteries that causes myocardial infarction and stroke, which are leading causes of morbidity and mortality worldwide^1^. Endothelial cell (EC) function and pro-inflammatory activation play a pivotal role at all stages of atherosclerosis, from initiation to plaque rupture and atherothrombotic complication^2^.

Intracellularly, a large number of atherogenic stimuli, like signals from growth factors, overnutrition, hyperglycemia and other stressors are integrated by the mechanistic target of rapamycin (mTOR)^3–5^. mTOR exists in 2 distinct complexes, mTORC1 and mTORC2, and its role as central regulator of cell growth and proliferation is well-established^3^. A growing body of evidence from preclinical studies demonstrates that genetic or pharmacological inhibition of mTORC1 suppresses atherosclerosis^6–15^ and promotes longevity in mice^16–18^. However, systemic mTORC1-inhibition in patients by rapamycin derivatives (rapalogs) is limited by adverse effects, of which hyperglycemia, insulin resistance and dyslipidemia are particularly troubling in the context of atherosclerosis^19,20^. Treatment-induced dyslipidemia may also explain why the net effect of pharmacological mTORC1-inhibition on cardiovascular risk in renal or liver transplantation patients was only neutral^21,22^.

Against this background, targeting of cell type-specific endogenous modulators of mTORC1 signaling like Proline-rich AKT substrate 40 kDa (PRAS40) may be a promising alternative. PRAS40 was identified as a component of the mTORC1 complex that negatively regulates mTORC1 signaling in a complex manner, which is only incompletely understood and, according to genetic evidence from Drosophila, highly cell type-dependent. This cell type-specific impact of PRAS40 on mTORC1 signaling may confer a therapeutic profile that is distinct from conventional mTORC1-inhibitors - possibly without the adverse effects that limit their clinical use in the context of atherosclerosis^23^. Indeed, we have previously demonstrated that PRAS40 gene therapy with adenoviral vectors improves hepatic insulin sensitivity and reduces systemic hyperglycemia and dyslipidemia in obese mice. Thus, in contrast to rapamycin and its derivatives, mTORC1-inhibition with PRAS40 appears to improve metabolic profile^24^.

However, the function of PRAS40 in distinct mammalian cell types, particularly endothelial cells, remains to be defined, and its role in vascular disease processes like atherosclerosis has not been investigated. Here we use *in vitro* loss-of-function and gain-of-function studies, as well as a conditional endothelial-specific PRAS40-knockout mouse model to investigate the endothelial function of PRAS40 in the context of atherosclerosis.

## Results

### PRAS40 Inhibits mTORC1 in Endothelial Cells

**Figure 1:**
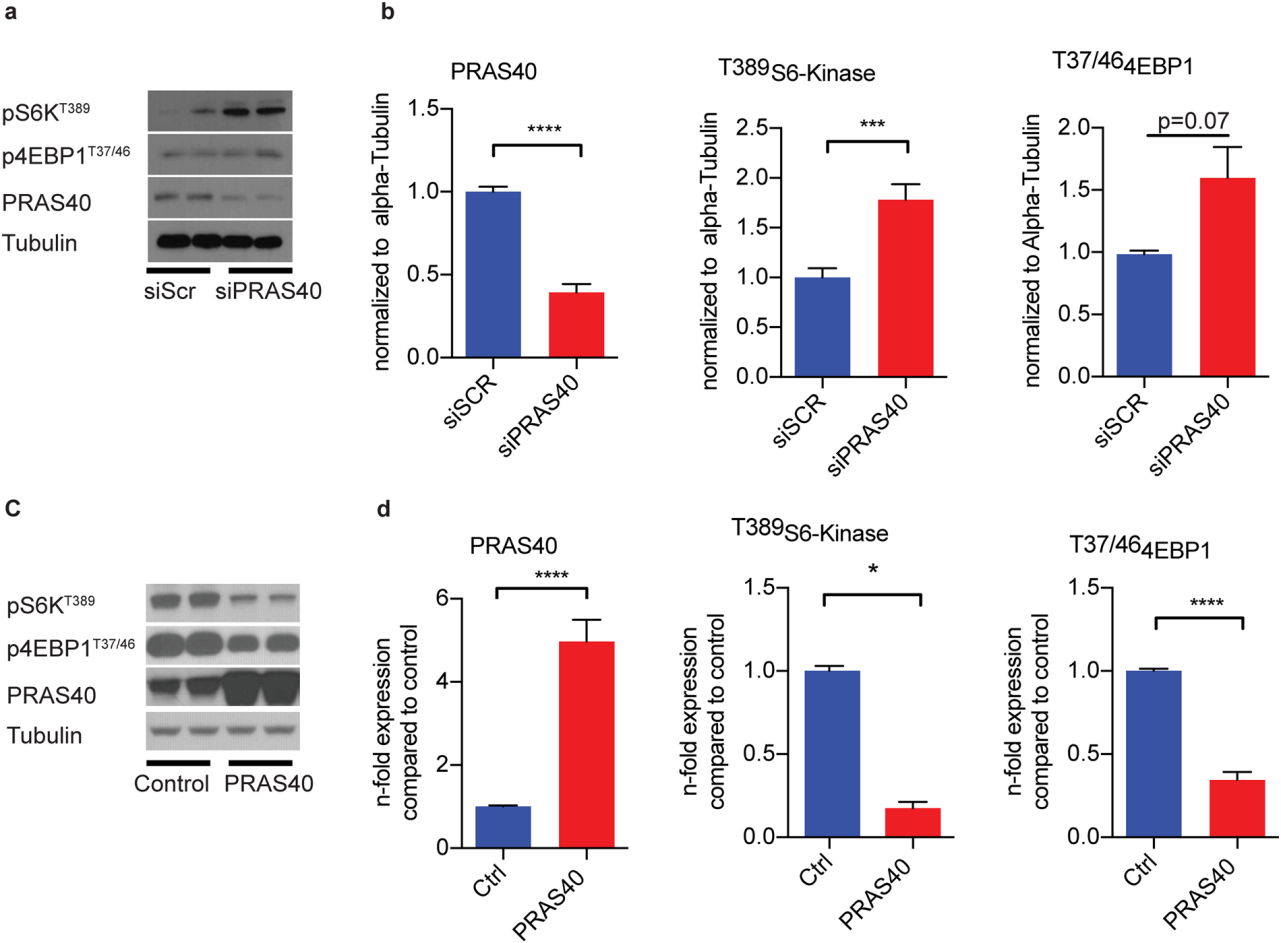
PRAS40 inhibits mTORC1 signaling in endothelial cells. (**a**,**b**) Representative immunoblot for indicated proteins after siRNA-mediated knockdown of PRAS40 in HUVECs and statistical analysis for indicated proteins based on analysis of 3 individual biological replicates. Data represent mean ± SEM; ***P < 0.001; ****P < 0.0001 (two-tailed student’s t-test). (**c**,**d**) Representative immunoblot for indicated proteins after adenoviral overexpression of PRAS40 in HUVECs and statistical analysis for indicated proteins based on analysis of 3 individual biological replicates. Data represent mean ± SEM; *P < 0.05; ****P < 0.0001 (two-tailed student’s t-test).

PRAS40 function has been shown to be highly cell type-dependent. Thus, in order to test its impact on mTORC1 signaling in endothelial cells, cultured human umbilical vein endothelial cells (HUVECs) were transfected with siRNA directed against PRAS40 or with scramble siRNA. mTORC1-mediated signaling was markedly enhanced by PRAS40 knockdown, as evidenced by increased phosphorylation of its downstream targets S6Kinase (S6K) and eukaryotic translation initiation factor 4E-binding protein 1 (4EBP1), respectively (Fig. 1a,b). In contrast, PRAS40 overexpression using recombinant adenoviruses inhibited mTORC1-mediated signaling in HUVECs (Fig. 1c,d). Thus, loss- and gain-of-function studies indicate a negative regulation of mTORC1 by PRAS40 in endothelial cells.

## PRAS40 Negatively Regulates Endothelial Pro-Inflammatoy Signaling

**Figure 2:**
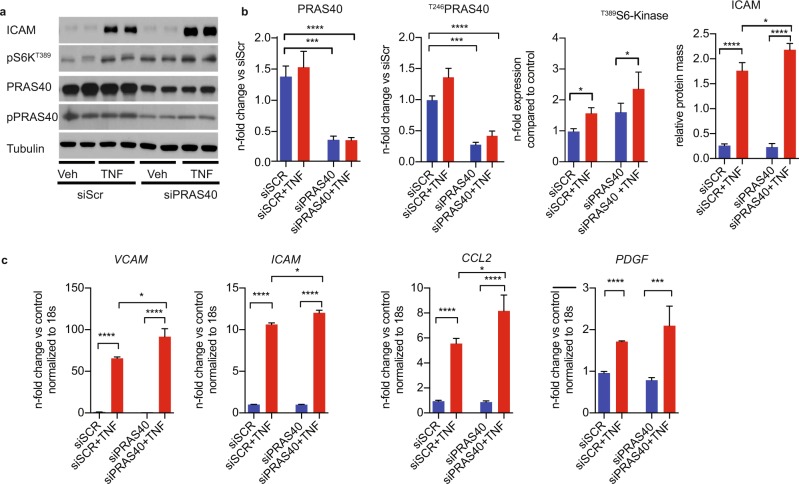
PRAS40 knockdown promotes pro-inflammatory signaling in endothelial cells. (**a**) Representative immunoblot for indicated proteins after siRNA-mediated knockdown of PRAS40 in HUVECs and treatment with TNFα (2,5 ng/ml). (**b**) Statistical analysis for indicated proteins based on analysis of 3 individual biological replicates. Data represent mean ± SEM; *P < 0.05; ***P < 0.001; ****P < 0.0001 (ANOVA followed by Bonferroni’s post-hoc comparisons). **(c)** Quantitative RT-PCR analysis for indicated transcripts after siRNA-mediated knockdown of PRAS40 in HUVECs and treatment with TNFα (2,5 ng/ml) based on analysis of 3 individual biological replicates. Data represent mean ± SEM; *P < 0.05; ***P < 0.001; ****P < 0.0001 (ANOVA followed by Bonferroni’s post-hoc comparisons).

**Figure 3:**
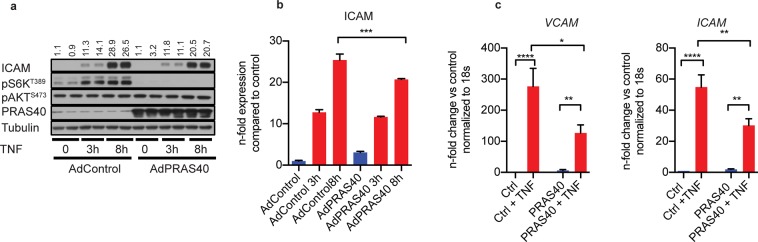
PRAS40 overexpression attenuates pro-inflammatory signaling in endothelial cells. (**a**) Representative immunoblot for indicated proteins after adenoviral overexpression of PRAS40 in HUVECs and treatment with TNFα (2,5 ng/ml) for indicated timepoints. (**b**) Statistical analysis for indicated proteins based on analysis of 3 individual biological replicates. Data represent mean ± SEM; ***P < 0.001. **(c)** Quantitative RT-PCR analysis for indicated transcripts after adenoviral overexpression of PRAS40 in HUVECs and treatment with TNFα (2,5 ng/ml) for 8 h based on analysis of 3 individual biological replicates. Data represent mean ± SEM; *P < 0.05; **P < 0.01; ****P < 0.0001 (ANOVA followed by Bonferroni’s post-hoc comparisons).

mTORC1 has been shown to regulate pro-inflammatory signaling as well as expression of atherogenic chemokines and leukocyte adhesion molecules like ICAM-1^25–27^. In order to elucidate the role of PRAS40 in the context of endothelial atherogenic signaling, we treated HUVECs with the pro-inflammatory cytokine tumor necrosis factor alpha (TNFα), which resulted in increased mTORC1 signaling and upregulation of ICAM-1 (Fig. 2a,b). Interestingly, siRNA-mediated knockdown of PRAS40 significantly augmented this TNFα-induced mTORC1 activation and upregulation of ICAM-1. Of note, PRAS40 knockdown also promoted TNFα-induced upregulation of other atherogenic molecules like VCAM-1 and CCL2, as determined by qRT-PCR (Fig. 2c). As specified by the given name, PRAS40 contains two proline-enriched stretches at the amino-terminus and an AKT consensus phosphorylation site (RXRXXS/T) located at Thr246. Phosphorylated PRAS40 dissociates from mTORC1 in response to growth factors, insulin, glucose and different nutrients and thereby releases the inhibitory function of PRAS40 on mTORC1. However, TNFα did not significantly induce phosphorylation of PRAS40 at threonine 256, suggesting that alternative mechanisms are involved in TNFα-induced activation of mTORC1. Still, siRNA-mediated knockdown of PRAS40 significantly augmented this TNFα-induced mTORC1 activation and upregulation of ICAM-1. In contrast, PRAS40 overexpression completely blocked TNFα-induced activation of mTORC1 and attenuated upregulation of the atherogenic leukocyte adhesion molecules ICAM-1 and VCAM-1 (Fig. 3). As previously published, PRAS40 specifically inhibited mTORC1 as determined by decreased phosphorylation of the canonical downstream target S6K, whereas phosphorylation of the mTORC2 downstream target AKT was not altered by PRAS40 overexpression.

## PRAS40 Attenuates Monocyte Recruitment by Endothelial Cells

**Figure 4:**
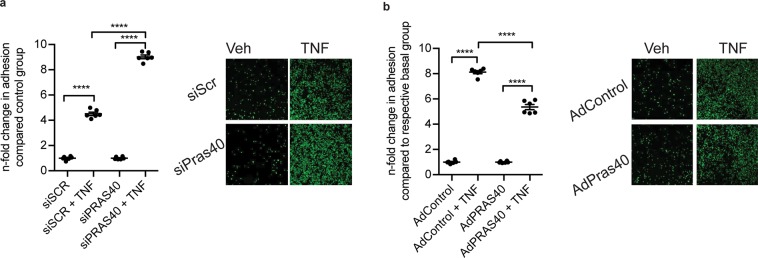
Endothelial PRAS40 regulates TNFα-induced monocyte adhesion. (**a**) Statistical analysis of fold changes in adhesion of mononcytes on HUVECs and representative images of monocyte adhesion after staining with Calcein AM after siRNA-mediated PRAS40 knockdown after treatment with TNFα. Data represent mean ± SEM; ****P < 0.0001. (**b**) Statistical analysis of fold changes in adhesion of mononcytes on HUVEC after adenoviral overexpression of PRAS40 after treatment with TNFα. Data represent mean ± SEM; ****P < 0.0001 (ANOVA followed by Bonferroni’s post-hoc comparisons).

In order to define the functional consequence of this negative regulation of mTORC1 activity and pro-inflammatory signaling by PRAS40, we tested its effect on monocyte recruitment to endothelial cells in a THP-1 monocyte adhesion assay. Consistent with the PRAS40-mediated suppression of leukocyte adhesion molecule- and chemokine-expression, PRAS40-knockdown strongly enhanced TNFα-induced recruitment of THP-1 monocytes (Fig. 4a). In contrast, PRAS40 overexpression markedly reduced THP-1 monocyte adhesion to TNFα-stimulated endothelial cells (Fig. 4b).

## PRAS40 Regulates Endothelial Proliferation and Apoptosis

As increased cell turnover is a hallmark of pro-inflammatory endothelial activation in the context of atherosclerosis, we investigated the impact of PRAS40 on endothelial proliferation. Consistent with the pro-inflammatory effects outlined above, knockdown of PRAS40 strongly promoted mitogenic effects of VEGF stimulation. In contrast, PRAS40 overexpression substantially reduced endothelial proliferation in response to VEGF (Supplementary Fig. S1).

Atherosclerosis-related increases in endothelial cell turnover are usually accompanied by enhanced apoptosis, and indeed, we also observed increased apoptosis rates upon stimulation with TNFα. PRAS40 overexpression strongly enhanced this effect, whereas apoptosis rates were markedly reduced by PRAS40-knockdown, indicating an anti-apoptotic effect of mTORC1 signaling in this context (Supplementary Fig. S2).

## Pharmacological mTORC1-Inhibition Mimicks the Effects of PRAS40 Overexpression

**Figure 5:**
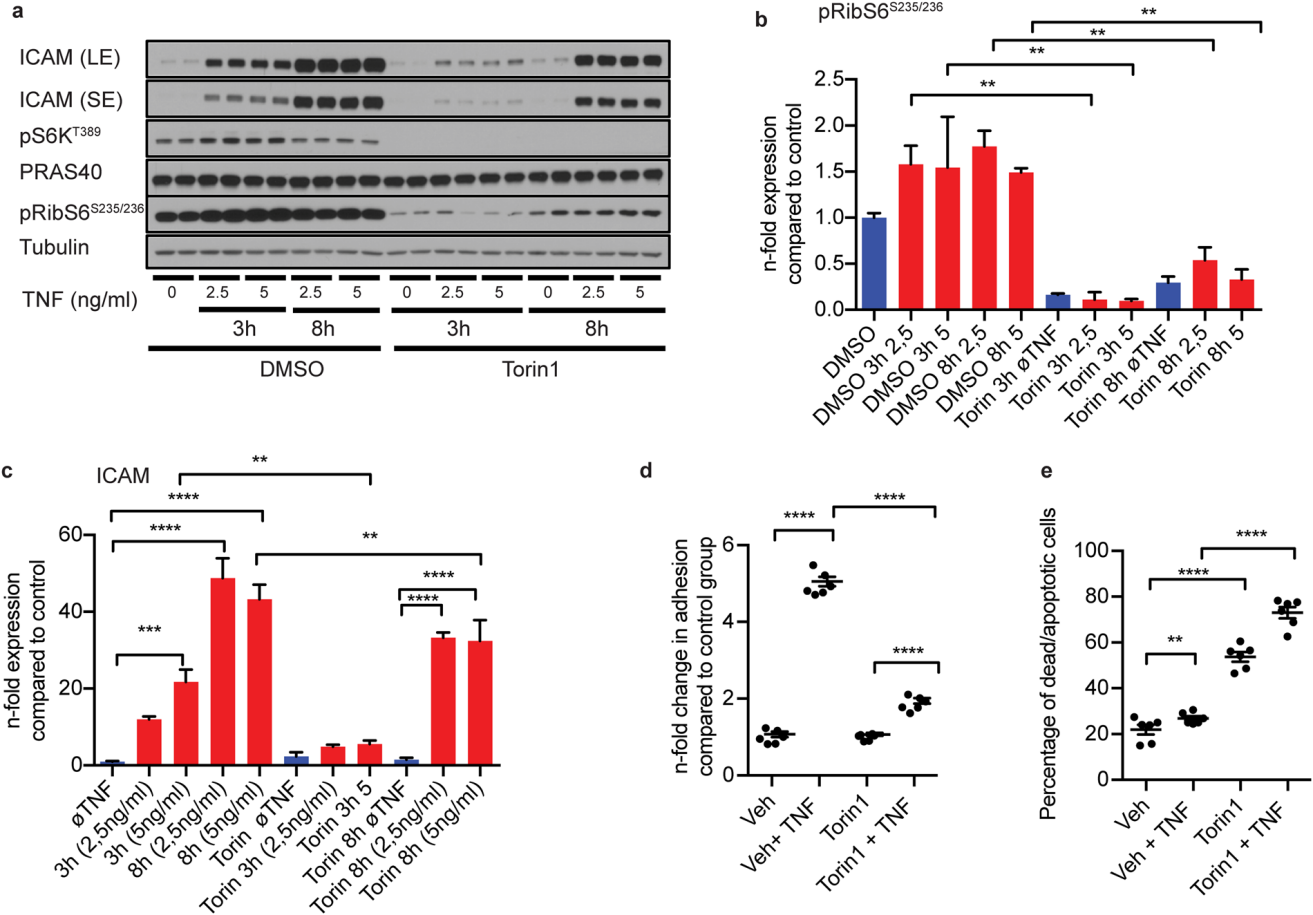
Pharmacological mTORC1-inhibition mimicks effects of PRAS40 overexpression in endothelial cells. (**a**) Representative immunoblot for indicated proteins after pharmacological mTORC1-inhibition with torin1 upon induction with indicated TNFα concentrations and timepoints. **(b,c)** Statistical analysis for indicated proteins based on analysis of 2 individual biological replicates. Data represent mean ± SEM; **P < 0.01; ***P < 0.001; ****P < 0.0001 (ANOVA followed by Bonferroni’s post-hoc comparisons). **(c)** Statistical analysis of fold changes in adhesion of mononcytes on HUVECs upon induction with TNFα and after treatment with torin1. Data represent mean ± SEM; ****P < 0.0001 (ANOVA followed by Bonferroni’s post-hoc comparisons). **(e)** Analysis of apoptotic HUVECs measured by flow-cytometry (annexin positive cells) after torin1 treatment and induction with TNFα (2,5 ng/ml) for 24 h based on the analysis of 3 individual biological replicates. Data represent mean ± SEM; **P < 0.01; ****P ≤ 0.0001 (ANOVA followed by Bonferroni’s post-hoc comparisons).

If PRAS40 indeed exerted its anti-atherogenic effects via negative regulation of mTORC1-mediated signaling, the effects of PRAS40 overexpression should be mimicked by pharmacological inhibition of mTORC1. Thus, we repeated key loss-of-function experiments using the novel mTOR-inhibitor torin1. mTORC1-mediated signaling, as reflected by phosphorylation of S6K and ribosomal S6 protein (RibS6), was completed blocked by torin1 (Fig. 5a,b). Of note, similar to PRAS40 overexpression, torin1-treatment suppressed endothelial upregulation of the leukocyte adhesion molecule ICAM-1 and monocyte adhesion to endothelial cells in response to TNFα (Fig. 5c,d). In addition, torin1 promoted endothelial apoptosis comparable to the pro-apoptotic effect of PRAS40 overexpression (Fig. 5e).

## Endothelial-Specific PRAS40 Deficiency Promotes Pro-Inflammatoy Signaling and Atherogenic Remodeling *in vivo*

Taken together, the *in vitro*-studies depicted above, indicate that PRAS40 inhibits atherogenic signaling and pro-inflammatory activation of endothelial cells by negative regulation of mTORC1. In order to validate these findings *in vivo* and to test their functional relevance in the context of atherosclerosis, we created a novel transgenic mouse line with conditional endothelial-specific PRAS40 deficiency, based on cre/loxP-mediated mutagenesis (EC-PRAS40-KO). In this respect we created mice with two loxP-flanked PRAS40-allels, and crossed them with mice harbouring a tamoxifen-inducible cre-recombinase under control of the endothelial-specific vascular endothelial cadherin (Cdh5)-promotor. Endothelium-specific PRAS40-deficiency was confirmed by immunostaining of carotid artery cross sections (Supplementary Fig. S3).

**Figure 6:**
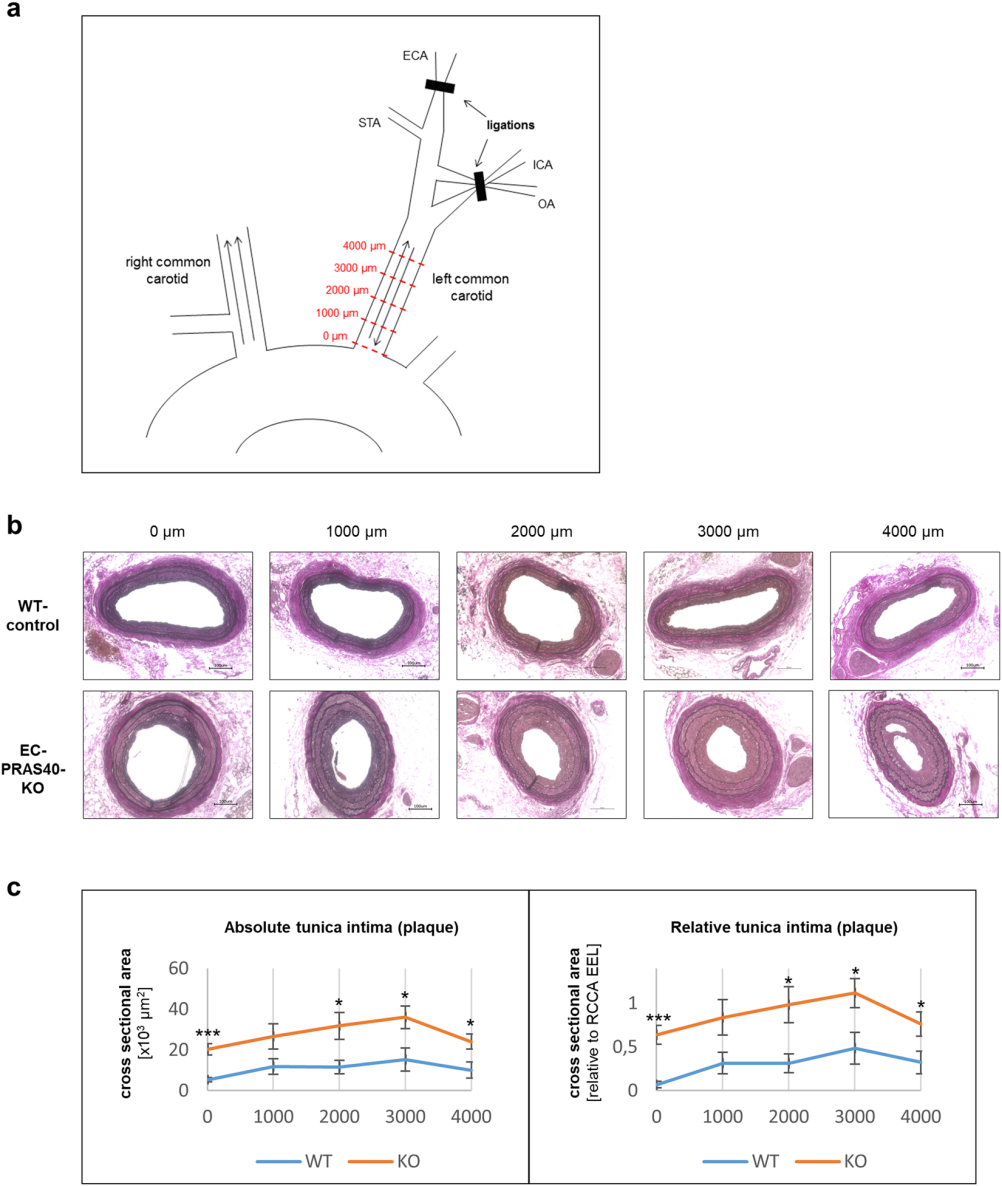
Endothelial-specific PRAS40 deficiency promotes atherogenic remodeling *in vivo*. (**a**) Schematic diagram of the partial carotid ligation. Arrows indicate direction of blood flow. Red dotted lines represent cross sections for quantitative analyses at indicated predefined distances from the proximal end of the vessel. ECA, external carotid artery; ICA, internal carotid artery; STA, superior thyroid artery; OA, occipital artery (**b**) Mice with tamoxifen-induced endothelium-specific PRAS40 deficiency (EC-PRAS40-KO) and tamoxifen-treated Cre-negative littermates (WT controls) were continuously exposed to western diet for a total of 10 weeks. Four weeks after partial ligation, left common carotid arteries as well as sham-operated right common carotid arteries were harvested for further analyses. Shown are representative carotid cross sections at five predefined distances from the proximal end of the carotid artery (0 µm, 1000 µm, 2000 µm, 3000 µm, 4000 µm) stained with elastic stain. Scale bars in black 100 µm. **(c)** Mean intimal cross sectional areas (including plaque and neointima) were quantified at each predefined distance (based on analysis of 4 different animals per genotype). Shown are absolute plaque areas as well as plaque areas in relation to the contralateral sham operated vessel size (area enclosed by external elastic lamina). Data represent mean ± SEM; *P < 0.05; **P < 0.01; ***P < 0.001 (two-tailed student’s t-test).

EC-PRAS40-KO mice and Cre-negative littermates were exposed to a model for disturbed blood flow and atherogenic vascular remodeling based on partial carotid ligation and western diet: Six weeks after tamoxifen-induced recombination and initiation of a western diet, all distal branches of the left common carotid artery were ligated, except for the superior thyroid artery, to establish disturbed but continuous blood flow as described by us previously (Fig. 6a)^28^. In combination with sustained western diet, this disturbed blood flow results in pro-inflammatory endothelial activation and atherogenic vascular remodeling in terms of neointimal hyperplasia and immune cell infiltration within four weeks^29^. Even though not observed consistently, initial atherosclerotic lesions with isolated foam cells and intracellular lipid accumulation can also develop.

**Figure 7:**
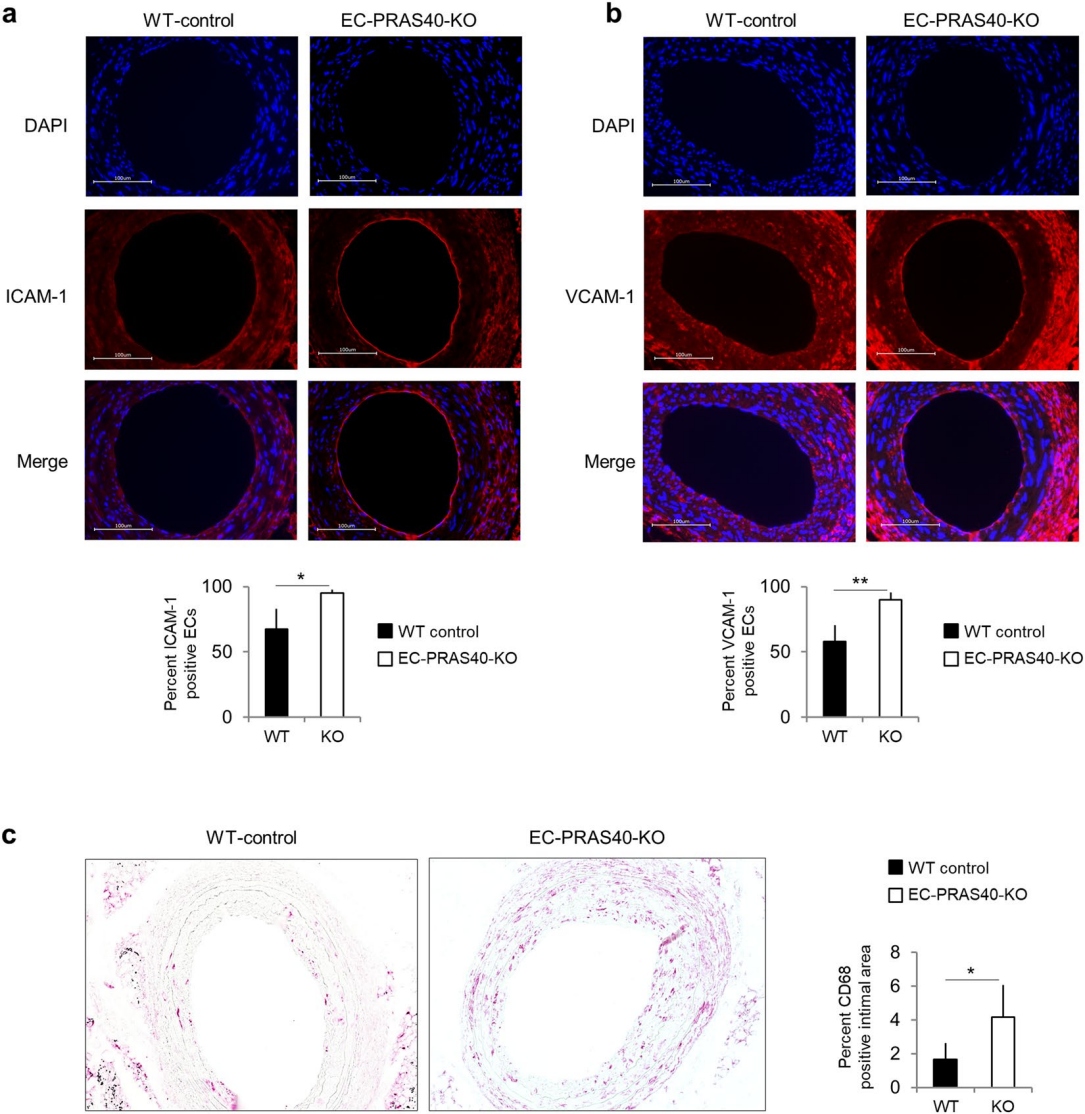
Endothelial-specific PRAS40 deficiency promotes pro-inflammatory signaling *in vivo*. (**a**,**b**) Shown are representative carotid cross section stained with DAPI (blue) and antibodies against the cell adhesion molecules ICAM-1 and VCAM-1, respectively (red). Scale bars in white 100 µm. Quantifications display percentage of endothelial cells defined by histomorphological features that stained positive for ICAM-1 and VCAM-1, respectively (based on analysis of 3 predefined sections each, from 4 different animals per genotype). (**c**) Shown are representative carotid cross section stained with an antibody against the macrophage marker CD68. Quantifications display the CD68-positive percentage of the intimal cross-sectional area (comprising plaque and neointima) based on analysis of 3 sections each, from 4 different animals per genotype. Data represent mean ± SEM; *P < 0.05; **P < 0.01 (two-tailed student’s t-test).

In line with our *in vitro*-data indicating an anti-atherogenic function of PRAS40, four weeks after partial carotid ligation, atherogenic remodeling and lesion development was strongly enhanced in EC-PRAS40-KO mice compared to littermate controls (Fig. 6b,c). Consistent with the concept of PRAS40-mediated inhibition of mTORC1-dependent pro-inflammatory signaling, deduced from our *in vitro* data, endothelial expression of the leukocyte adhesion molecules ICAM-1 and VCAM-1 was markedly enhanced in EC-PRAS40-KO mice (Fig. 7a,b). In line with this increased expression of cell adhesion molecules, macrophages were more abundant in intimal lesions of EC-PRAS40-KO mice than in littermate controls (Fig. 7c).

## Discussion

mTORC1-mediated signaling is activated under atherogenic conditions or in the context of atherosclerosis, and inhibition of mTORC1 has been demonstrated to reduce atherosclerotic plaque development through pleiotropic effects in a large number of preclinical studies^6–15^. Moreover, mTORC1-inhibitors like rapamycin or its derivatives are known to promote longevity in mice^16–18^. However, the systemic use of mTORC1-inhibitors in patients is limited by adverse effects, of which hyperglycemia, insulin resistance and dyslipidemia are particularly troubling in the context of atherosclerosis^20^. In fact, the significant treatment-induced dyslipidemia may also explain why, despite the promising preclinical data, mTORC1-inhibitors had only a neutral net effect on cardiovascular risk in renal or liver transplantation patients^21,22^. In that respect it is noteworthy, that the above-mentioned preclinical studies were largely based on genetic atherosclerosis models resulting in excessive hypercholesterolemia. Under these conditions moderate increases in cholesterol levels elicited by mTORC1-inhibition may not come into effect.

In this study, we investigated the endogenous mTORC1-modulator PRAS40 as a potential target for the treatment of atherosclerosis. Due to its highly cell type-specific impact on mTORC1 signaling, PRAS40-mediated inhibition of mTORC1 may offer a distinct therapeutic profile compared to conventional mTORC1-inhibitors - possibly with less adverse effects. Indeed, while rapamycin and its derivatives detrimentally affect lipid and glucose homeostasis^20^, we have previously demonstrated that PRAS40 gene therapy with adenoviral vectors improves hepatic insulin sensitivity and reduces systemic hyperglycemia and hyperlipidemia in obese mice^24^.

However, the specific function of PRAS40 in endothelial cells and a potential role in atherosclerosis have not been investigated so far. The *in vitro* gain-of-function- and loss-of-function-studies presented here, show that PRAS40 negatively regulates mTORC1 in endothelial cells, at the same time attenuating endothelial inflammatory activation and monocyte recruitment in response to atherogenic stimuli. These findings are consistent with a number of previous reports demonstrating mTORC1-dependent pro-inflammatory signaling in various cell types^26,27,30^. Moreover, systemic mTORC1-inhibition by rapamycin was consistently shown to reduce chemokine expression and monocyte recruitment in experimental atherosclerosis^6–8,12,15^. Even though mTORC1 function in this respect has not been specifically defined in endothelial cells, individual reports have suggested an mTORC1-dependent endothelial regulation of atherogenic molecules like VCAM-1 or endothelin-1^31^. The precise mechanisms of mTORC1-mediated pro-inflammatory signaling remain to be elucidated. However, recently a non-canonical pathway for the regulation of chemokine expression through mTORC1-mediated dephosphorylation of transcription factor forkhead box K1 (FOXK1) has been identified^30^.

Pro-inflammatory endothelial activation and endothelial monocyte recruitment are critical events in atherosclerotic lesion development. In line with our *in vitro* findings, endothelial-specific PRAS40-deletion in mice resulted in strongly increased atherogenic remodeling and lesion formation in our murine model of partial carotid ligation and western diet. These observations are consistent with numerous reports demonstrating anti-atherosclerotic effects of pharmacological mTORC1-inhibition in mice. However, it has to be considered that, unlike our murine disease model eliciting only atherogenic remodeling and initial atherosclerotic lesions, these studies investigated more advanced stages of atherosclerosis in atheroprone mouse strains lacking the LDL receptor or the apolipoprotein E. While these genetic mouse models may better mimic the complete picture of human atherosclerosis, the lifelong defect in lipid homeostasis resulting in excessive hypercholesterolemia might also be a confounding factor^29^. Indeed, findings from previous studies suggest that mTORC1-inhibition is less effective in late stages of atherosclerosis and that potential effects are obscured by the excessive hypercholesterolemia in genetic atherosclerosis models^7^.

Consistent with the anti-inflammatory effects of PRAS40 overexpression in our *in vitro* experiments and the above-mentioned studies on pharmacological mTORC1-inhibition, endothelial expression of the leukocyte adhesion molecules ICAM-1 and VCAM-1 was markedly enhanced in EC-PRAS40-KO mice and coincided with higher numbers of immune cells in the respective intimal lesions. These data suggest that PRAS40 exerts its atheroprotective function, at least partially, through the suppression of endothelial pro-inflammatory activation. However, besides its anti-inflammatory effects, we also found PRAS40 to inhibit endothelial proliferation, which is in line with well-established effects of mTORC1 signaling on cell cycle-control in various cell types^3^. These anti-mitogenic effects of PRAS40 might contribute to its atheroprotective function, too, and suppression of endothelial proliferation has even been proposed to prevent plaque destabilization^11^.

While the anti-inflammatory and anti-proliferative effects of PRAS40 can be readily reconciled with its atheroprotective function in endothelial cells, the positive regulation of apoptotic signaling may appear conflictive with this concept. First of all, our observations of PRAS40-mediated pro-apoptotic effects are in line with a large body of evidence demonstrating that mTORC1 is capable of regulating apoptosis through multiple downstream targets including apoptosis-regulatory proteins such as p53, Bad and Bcl-2, in a cell type- and context-dependent manner^32^. Interestingly, both pro- and anti-apoptotic effects of mTORC1 have been reported in endothelial cells^33,34^. The fact that atheroprone vascular regions are characterized by increased endothelial proliferation and apoptosis rates may indeed be suggestive of a mechanistic link between endothelial cell turnover and the susceptibility to atherosclerotic plaque development. However the role of endothelial apoptosis in atherosclerotic lesion development is probably more complex and only incompletely understood^35^.

After all, the atheroprotective effects of PRAS40 may be pleiotropic, and the relative contributions of its anti-inflammatory, anti-proliferative and pro-apoptotic effects in endothelial cells cannot be precisely defined. Moreover, we cannot resolve, whether the atheroprotective effects of PRAS40 are indeed exerted via inhibition of mTORC1 signaling. However, the fact that the effects of PRAS40 overexpression were mimicked by pharmacological mTORC1-inhibition is suggestive of such causality.

Thus, based on our studies, we hypothesize that PRAS40 suppresses development of atherosclerosis, at least in part, via inhibition of mTORC1-mediated pro-inflammatory signaling in endothelial cells. In conjunction with its favourable effects on metabolic homeostasis, the overall therapeutic profile of PRAS40-treatment appears to be beneficial, compared to conventional mTORC1-inhibitors, the systemic use of which is limited by off-target effects including hyperglycemia, insulin resistance and dyslipidemia. Taken together, PRAS40 may qualify as a potential therapeutic target for the treatment of atherosclerosis.

## Materials and Methods

### Reagents

Rapamycin, diluted and stored in DMSO, was purchased from Santa Cruz Biotechnology (sc-3504B). Torin 1, diluted and stored in DMSO, was from Cayman Chemical Company (Iten#10997). TNFα, diluted and stored in PBS, was bought from PeproTech (35-01A, LOT No. 121054-1). Protease and phosphatase inhibitors were purchased from Roche. β-Mercaptoethanol and DMSO were purchased from Sigma-Aldrich, Tween 20 detergent from Carl Roth.

### Cell culture

PBS and RPMI 1640 medium were purchased from Sigma-Aldrich, FBS from Merck Milipore. Penicillin, streptomycin & L-glutamine (100x) (PSG), MEM Non-Essential Amino Acids Solution (100x), Trypsin/EDTA solution, HEPES buffering agent were from Life Technologies.

Human umbilical vein endothelial cells (HUVECs) were purchased from Lonza and used as a model of endothelial cell function. HUVECs were cultivated in Endothelial Cell Growth Medium-2 (EGM-2) (Lonza) containing the Endothelial Basal Medium-2 (Lonza), 2% FBS and other growth factors included in the kit by Lonza. HUVECs were cultivated and normally used for experiments at passage 5–6. HUVECs were cultivated to a maximal confluency of 70–90%. Then a subculturing process was performed, or they were plated into 6-well plates for experiments. For reseeding, the cells were washed with PBS and detached using trypsin/EDTA solution at 0.025%. For experiments, the endothelial cells, frozen in cryo vials at passage 4, were thawed, cultivated and re-plated in 6-well plates at 80,000–120,000 cells per well in EBM-2 medium supplemented with 10% FBS. After 24 hours, the medium was changed to EBM-2 with 5% FBS to conduct transfection, infection or direct experiments. For cryopreservation, special media was mixed consisting of 80% EGM-2, 10% DMSO and 10% FBS.

The THP-1 monocytes kindly provided by AG Leuschner (University Hospital Heidelberg) belong to a cell line derived from an acute monocytic leukemia patient. These suspension cells were cultivated in a medium mixture consisting of RPMI 1640 culture medium, 10% FBS, 10 mM HEPES buffering agent, 50 µM β-Mercaptoethanol and supplemented with non-essential amino acids and an antibiotic solution of penicillin (100IE/ml), streptomycin (100 µg/ml) and L-glutamine (292 µg/ml) (PSG). A concentration of 300,000–800,000 cells/ml was allowed before subculturing was conducted. To verify cell number, medium color was checked daily and cell number was determined periodically using a counting chamber. The medium for cryopreservation consisted of 76.5% RPMI 1640, 18.5% FBS and 5% DMSO supplemented with PSG.

### Western blotting

HUVECs were washed with ice-cold PBS and lysed by a protein lysis buffer (20 mM Tris, 150 mM NaCl, 1% Triton X-100, 0.1% SDS, protease and phosphatase inhibitor). To determine the protein concentration, the DC Protein Assay Kit kit (Bio-Rad), based on the Lowry method, was used according to the manufacturer’s instructions. If necessary, the protein samples were frozen at −20 °C before further processing. After isolation and the concentration assay, the loading samples were prepared to have a desired protein mass, which ranged between 10–15 µg; the rest of the loading sample consisted of Laemmli Sample Buffer (Bio-Rad) supplemented with 10% β-Mercaptoethanol and the protein lysis buffer. The loading samples were heated for 5 minutes at 95 °C to ensure denaturation. The lysates were resolved using SDS-gels (Bio-Rad #3450118, #3450123) in an electrophoresis chamber (Bio-Rad). Afterwards, the proteins were blotted onto a PVDF membrane (Merck Millipore). For the immunostaining process, membranes were washed with Tris-buffered saline with Tween 20 (TBST) and blocked at room temperature for at least 1 hour using I-Block (Life Technologies) dissolved in TBST. PVDF membranes were then incubated at 4 °C overnight with the respective primary antibodies diluted in blocking reagent and TBST buffer. The proteins targeted by the primary antibodies used were used in the following dilutions:

- Intercellular adhesion molecule 1 – ICAM-1, 1:25 000; Santa Cruz Biotechnology (sc8439)
- Phospho-p70 S6 Kinase (Thr389) – pS6K1, 1:1000; Cell Signaling Technology (#9205)
- Proline-rich Akt substrate of 40 kDa – PRAS40, 1:10 000–1:5000; Cell Signaling Technology (#2691)
- Phospho-Proline-rich Akt substrate of 40 kDa (Thr246) – pPRAS40, 1:10000–1:5000
- Phospho-Akt (Ser473) – pAkt, 1:1000; Cell Signaling Technology (#9271)
- Phospho-S6 Ribosomal Protein (Ser235/236) – pRPS6, 1:5000; Cell Signaling Technology (#2211)
- Phospho-4EBP1 (S65) – p4EBP1, 1:5000; Cell Signaling Technology (#9451)
- α-Tubulin –1:20 000, Cell Signaling Technology (#2144S)

The detection of the proteins was performed using horseradish peroxidase conjugated secondary antibodies, Western Lightning Plus-ECL (Perkin Elmer) and CL-XPosure Films (Thermo Fisher Scientific). Utilizing an open source image processing software called Fiji, based on ImageJ, the width and the intensity of each band was determined as a numerical value. The bands of the protein α-Tubulin served as control.

### RNA interference

siRNA dependent RNA interference was utilized in silencing the AKT1S1 gene encoding the protein PRAS40. The siRNAs used were purchased from Thermo Fisher Scientific: Negative Control siRNA (Silencer Negative Control No. 2 siRNA – #AM4613) and AKT1S1-targeting siRNA (siRNA ID s38945, #4392420). The transfection of the siRNAs was performed using the reagent lipofectamine 2000 (Thermo Fisher Scientific) according to the following protocol based on the manufacturer’s instructions. For each well

- a dilution of siRNA in 125 µl of EBM-2 resulting in an siRNA concentration of 500 nM was performed.
- 2,5 µl of the transfection reagent was diluted in 125 µl of EBM-2
- both reagents were mixed and incubated for 5 minutes
- the two mixtures were combined and incubated for 20 minutes

The medium of the cells to be transfected was changed to EBM-2 supplemented with 5% FBS and the prepared siRNA complexes were added to each well. This resulted in a final siRNA concentration of 50 nM and a well volume of 1,25 ml. The cells were exposed to the transfecting medium for 24 hours.

### Adenoviral overexpression of PRAS40

Both viruses PRAS40 Wildtype-Adenovirus and Control-Adenovirus were generated using the Gateway cloning system^36^. Normally stored frozen in viral storage buffer (20 nM Tris/HCL, 25 mM NaCl, 2,5% Glycerol, pH adjusted to 8.0 at 22 °C), the viral particles were added to the EBM-2 medium with 5% FBS to the desired multiplicity of infection (MOI, number infectious particles per cell). The cells were exposed to the viral medium for 24 hours.

### Adhesion assay

For the PRAS40 modulation experiments, HUVECs were either transfected with siRNA or infected by adenovirus as described above. The day following the transfection or infection, the cells were harvested from the 6-well plates and seeded in 96-well plates with a concentration of 50,000 cells and 100 μl of EBM-2 supplemented with 10% FBS. For the torin experiments, HUVECs were cultivated parallelly. After reseeding, the cells were given 48 hours to reattach and form a complete monolayer. HUVECs were stimulated with TNFα, diluted in PBS, at a final concentration of 2,5 ng/ml, torin 1 was used at a concentration of 150 nM for 3 hours. The THP-1 monocytes were stained and prepared using eBioscience Calcein AM Viability Dye (Thermo Fisher Scientific), a cell permeant dye staining metabolically active and live cells, according to the Vybrant Cell Adhesion Assay Kit’s manual (Thermo Fisher Scientific, #V13181). The stained THP-1 monocytes were added to the HUVEC wells at a number of 500 000/well for adhesion and the non-adhering cells were removed by washing. Utilizing a plate reader, possessing a FITC filter set, the fluorescence of each well was measured at 9 different locations after excitation. The mean of each well was used for further data processing.

### Apoptosis

Apoptosis was induced by treating the HUVECs with TNFα at 120 ng/ml for 24 hours in EBM-2 supplemented with 5% FBS. Simultaneously to the addition of TNFα, for the mTOR inhibitor experiments rapamycin and torin 1 were added. After 24 hours treatment was complete and a flow-cytometry (BD FACSVerse) dependent assay using the Dead Cell Apoptosis Kit with Annexin V FITC and PI (Thermo Fisher Scientific, #V13242) was performed.

### Proliferation

For siRNA-mediated knockdown or adenoviral overexpression of PRAS40, HUVECs were first cultivated, transfected and infected as described above. Then the cells were harvested and from the 6-well plate and reseeded in special imaging 96-well plates (Corning) at desired concentration of approximately 1000 cells per well. On day 0, the cells were then given approximately 4 hours to attach to the plate after reseeding to the 96-well plates. During the attachment phase, the cells were given medium containing 10% FBS to facilitate the process. Afterwards, the initial count was performed using the CyQuant Direct Cell Proliferation Assay Kit. (Thermo Fisher Scientific, # C35011). For the counting process itself, pictures were automatically taken, processed and counted digitally using the In Cell Analyzer 2200 and its software. This allowed cell count determination in absolute numbers. For the comparison of cell proliferation over the time course of 3 days, cells were cultivated in 5% FBS EBM-2 and either stimulated with VEGF (kindly provided by AG Hassel (University Hospital Heidelberg) at a concentration of 50 ng/ml. Medium change including the re-addition of VEGF was performed on day 2 (48 hours after seeding) of each trial. Measurement of cell count was performed every 24 hours after initial cell number determination.

### Genetic mouse model

To study PRAS40 *in vivo*, a novel PRAS40 knockout mouse line was created with tamoxifen-inducible endothelial-specific PRAS40 deficiency. An embryonic stem cell clone (EPD0156_2_A05-European Conditional Mouse Mutagenesis Program) was used to generate mice with loxP-flanked PRAS40 allels. Endothelial-specific knockout mice were generated by crossing floxed PRAS40 mice with Cdh5(PAC)-CreERT2 mice^37^. Cre^+/−^;Pras40^fl/fl^ mice were considered knockout animals (EC-PRAS40-KO), whereas Cre-negative littermates were considered wildtype controls.

### Mouse model of atherogenic remodeling

From six weeks of age, mice were on continuous western diet (Ssniff^®^ TD88137, 0,3% cholesterol) for a total of 10 weeks. At 7 weeks of age Cre recombinase was activated in EC-PRAS40-KO mice by intraperitoneal injections of tamoxifen (Sigma, T5648, 1 mg/animal/day on five consecutive days). Cre-negative littermate control mice underwent the same procedure.

At 12 weeks of age partial carotid ligation was performed as described by us before^28^. Briefly, mice were anaesthetized by intraperitoneal injection of ketamine (120 mg/kg, Pfizer, Germany) and xylazine (16 mg/kg, Bayer; Germany) and placed on a heated surgical pad. After hair removal a midline cervical incision was made and the distal branches of the left common carotid artery were exposed. Three of the four branches (external carotid artery, internal carotid artery and occipital artery) were then ligated distal to the superior thyroid artery branch off, which was left open as an outflow (see schematic diagram in Fig. 6a). 6.0 silk sutures (Serag-Wiessner) were used for ligations; the skin was sutured with absorbable 6.0 silk suture (CatGut, Germany). After surgery, animals were monitored in a chamber on a heating pad until recovery. Western diet was continued until harvesting of carotid arteries 4 weeks after partial carotid ligation.

All animal experiments were approved by the animal ethics committee of the federal state of Berlin (Tierversuchskommission des Landes Berlin, Germany) and were performed accordingly under adherence to the effective guidelines and the German Protection of Animals Act.

### Histology and immunohistochemistry

For histology, vessels were perfused and fixed in 4% PFA overnight. Vessels were then rehydrated using increasing concentrations of ethanol and embedded in paraffin. Paraffin-embedded arteries were cut in 5-μm serial cross sections mounted on slides.

For morphometric analyses, slides were rehydrated and stained using a Modified Verhoeff Van Gieson Elastic Stain Kit (Sigma Aldrich HT25) according to the manufacturer’s instructions. In partially ligated left common carotid arteries, cross sections from five predefined distances (0 μm, 1000 μm, 2000 μm, 3000 μm and 4000 μm, respectively) from the proximal end of the carotid artery were analysed (see schematic diagram in Fig. 6a). Photoshop CS5 extended software (Adobe) was used to measure circumferences of internal elastic lamina, external elastic lamina, and lumen, as well as medial, intimal, and luminal cross-sectional areas as described by us previously^38^. For relative analyses plaque areas were quantified in relation to the cross-sectional area of the sham-operated contralateral carotid as determined by the area enclosed by the external elastic lamina.

For immunohistochemistry, paraffin sections were rehydrated, and subsequently boiled for 3 minutes in citrate buffer (pH = 6) in a microwave oven for epitope retrieval. Epitopes were then blocked in PBS supplemented with 5% normal goat serum and 1% BSA for 30 minutes. Slides were incubated with primary antibodies (1:200 dilution in blocking buffer) at 4 °C overnight. Primary antibodies were then detected with a biotinylated secondary antibody to rabbit IgG followed by incubation with avidin–biotin complex (AK-5000, Vector Laboratories) according to manufacturer’s instructions. Staining was then visualized using VectorRed-Kit (SK-5100, Vector Laboratories) following the manufacturer’s instructions. Finally, slides were mounted using DAPI-containing aqueous mounting medium (H-1200, Vector Labotatories).

### Antibodies

Anti-VCAM1: ab134047 (Abcam), anti-ICAM1: ab119871 (Abcam), anti-CD68: ab125212 (Abcam), anti-rabbit IgG: BA-1000 (Vector Laboratories), anti rat-IgG: BA-9401 (Vector Laboratories).

For quantifitative analyses, the percentage of positively stained endothelial cells (ICAM-1- and VCAM-1-stainings) or the percentage of positively stained intimal area (CD68 staining) was calculated. Of note, endothelial cells were defined based on characteristic histomorphological features (cells with thin elongated nuclei constituting the luminal lining of the vessel). A minimum of three cross sections from the proximal, medial and distal part of the vessel were analysed and a vessel mean calculated.

### Microscopy

Microscopic images were captured using the Keyence HS all-in-one BZ 9000 microscope. For fluorescence images, the CFI Plan Apo λ 40x lens was used. The images have a resolution of 1360 × 1024 pixels, while the scaling is 3,75 pixels/µm. Capture time was 1/12 s for DAPI and 1/10 s for VectorRed. Colors are displayed as pseudocolors using blue for fluorescence in the DAPI channel and red for Fluorescence in the VectorRed channel. The used fluorophores have the following excitation/emission wavelengths: DAPI: 360 nm/460 nm; VectorRed: 365–560 nm/ > 560 nm. Images were captured using the following filter sets: DAPI 340/360 nm Excitation - 450/460 Emission; VectorRed: 513/556 Excitation – 570/613 Emission.

For images of elastica and CD68 stainings, the CFI Plan Apo λ 20x lens was used. These images have a resolution of 4080 × 3072 pixels, while the scaling is 5,63 pixels/µm. Capture time was 1/30 s, bit-depth was 24.

### Statistics

For statistical analysis, PRISM 6 software by GraphPad Software was utilized. Comparisons in experiments of more than two groups were performed with one-way ANOVA without matching or pairing with multiple comparisons being performed using preselected groups of interest. In the diagrams, the values presented are the arithmetic means of the group with the error bars indicating the standard error of the mean (SEM). If a comparison was performed between two groups unpaired t-test was utilized to determine significant difference.

## Supplementary information

Supplementary data

## Data Availability

The datasets generated during and/or analysed during the current study are available from the corresponding author on reasonable request.
