# Supplementary material for: PRAS40 suppresses atherogenesis through inhibition of mTORC1-dependent pro-inflammatory signaling in endothelial cells
Source: Sci Rep. 2019 Nov 14;9:16787. doi: 10.1038/s41598-019-53098-1 (PMC6856095; doi:10.1038/s41598-019-53098-1)
Supplement: Supplementary file 1 — Supplementary data [file 41598_2019_53098_MOESM1_ESM.pdf]

Supplementary data

PRAS40 suppresses atherogenesis through inhibition of mTORC1-dependent pro-inflammatory signaling in endothelial cells

Kevin Sun Zhang, Johannes Schecker, Alexandros Krull, Eva Riechert, Lonny Jürgensen, Verena Kamuf-Schenk, Jana Burghaus, Leon Kiper, Thanh Cao Ho, Kerstin Wöltje, Verena Stangl, Hugo A. Katus, Karl Stangl, Mirko Völkers, and Till F. Althoff

a

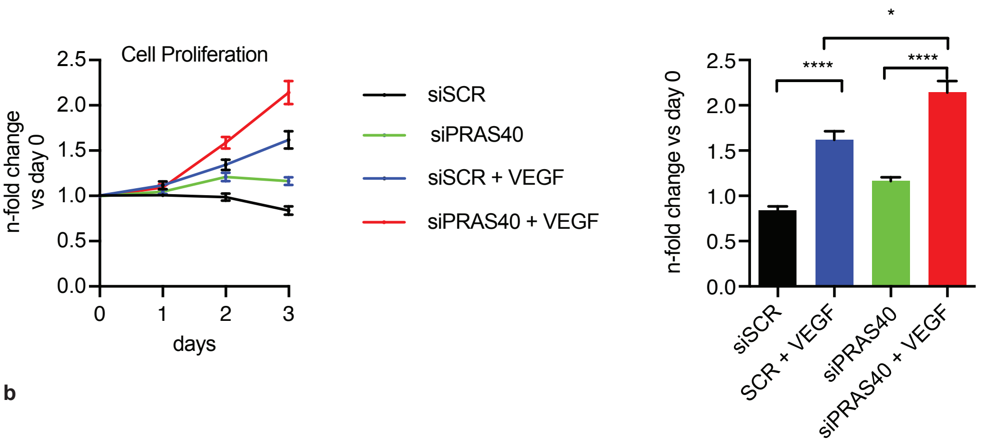

b

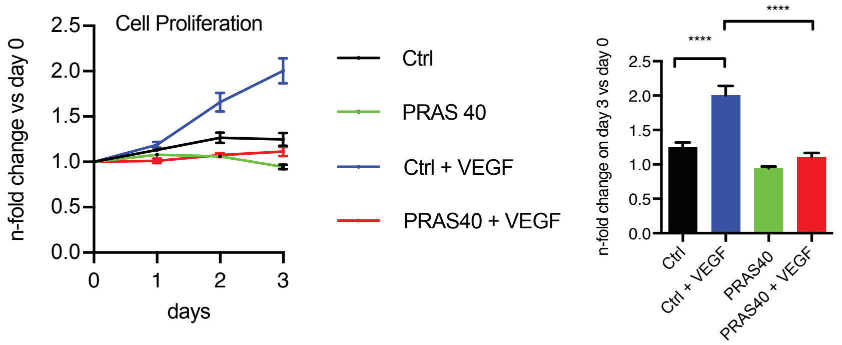

Supplementary Figure S1. PRAS40 regulates proliferation ofendothelial cells.
(a) PRAS40 knockdown promotes proliferation of HUVECs upon VEGF treatment for indicated timepoints (50 ng/ml).
n=3 in triplicates each experiment. Data represent mean ± SEM; * P≤0.05, **** P≤0.0001 (ANOVA followed by Bonferroni's post-hoc comparisons).
(b) PRAS40 overexpression blocked VEGF-induced proliferation of HUVECs (50 ng/ml). n=3 in triplicates each experiment.
Data represent mean ± SEM; **** P≤0.0001 (ANOVA followed by Bonferroni's post-hoc comparisons).

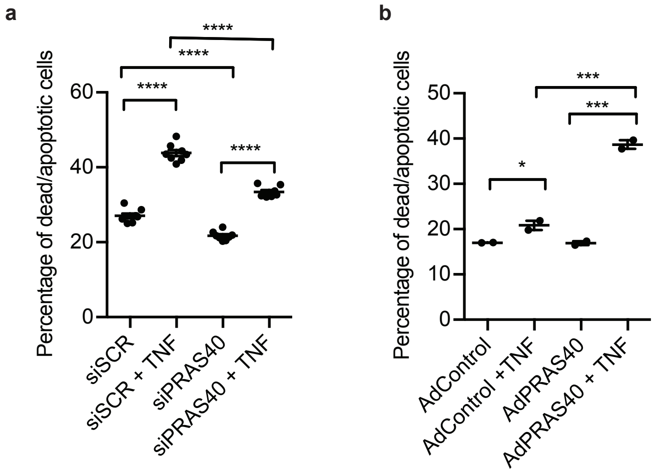

Supplementary Figure S2. PRAS40 dependent regulation of apoptosis in endothelial cells.
(a) Cell death quantified after siRNA mediated knockdown by flow cytometric detection of annexin staining upon TNFα treatment for 24h based on analysis of 3 individual biological replicates. Data represent mean ± SEM;
** P P≤0.05, **** P≤0.0001 (ANOVA followed by Bonferroni's post-hoc comparisons).
(b) Cell death quantified after adenoviral overexpression of PRAS40 by flow cytometric detection of annexin staining upon TNFα treatment for 24h based on analysis of 3 individual biological replicates. Data represent mean ± SEM;
** P P≤0.05, *** P≤0.001 (ANOVA followed by Bonferroni's post-hoc comparisons).

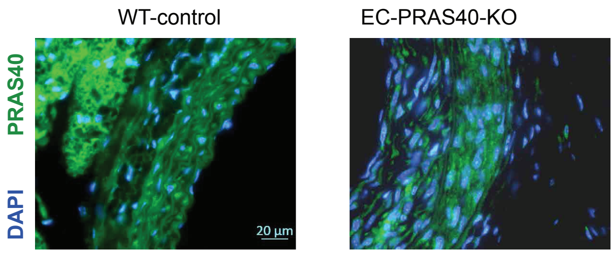

Supplementary Figure S3. Endothelial-specific PRAS40 knockout *in vivo*.
Immunohistochemistry showing reduced endothelial PRAS40 expression in EC-PRAS40-KO vessels (right) compared to vessels of littermate controls. Blue: DAPI; green: PRAS40; scale bar 20uM.

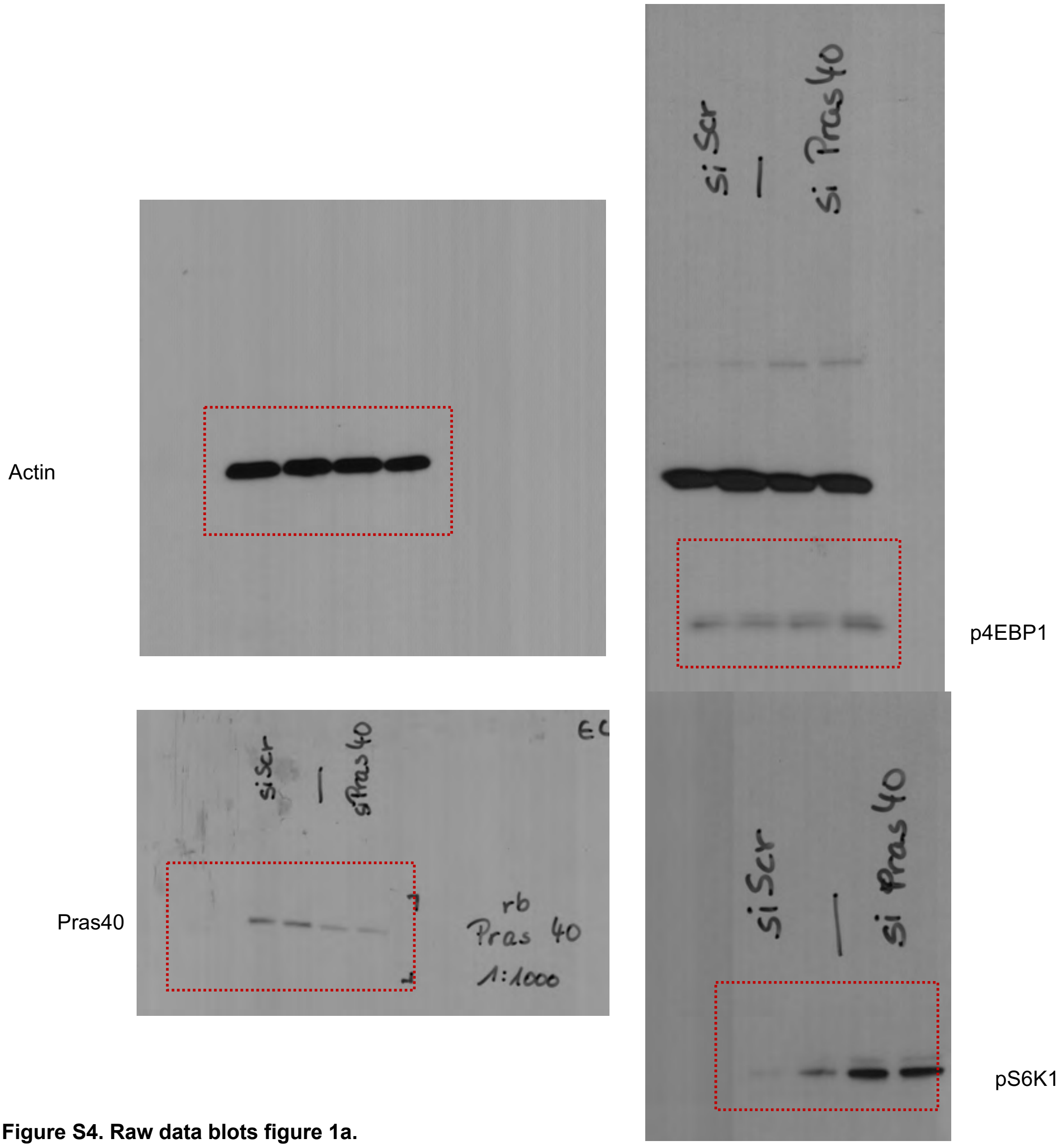

Supplementary Figure S4. Raw data blots figure 1a.

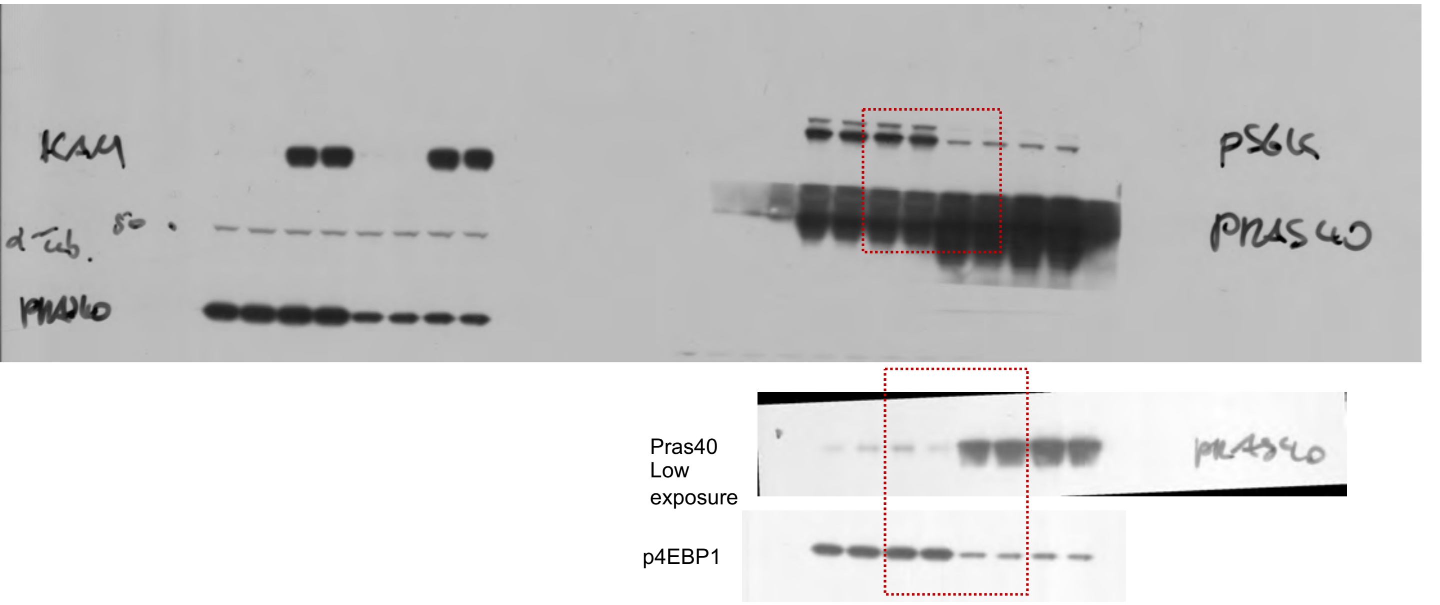

Supplementary Figure S5. Raw data blots figure 1c

Blots Figure 2

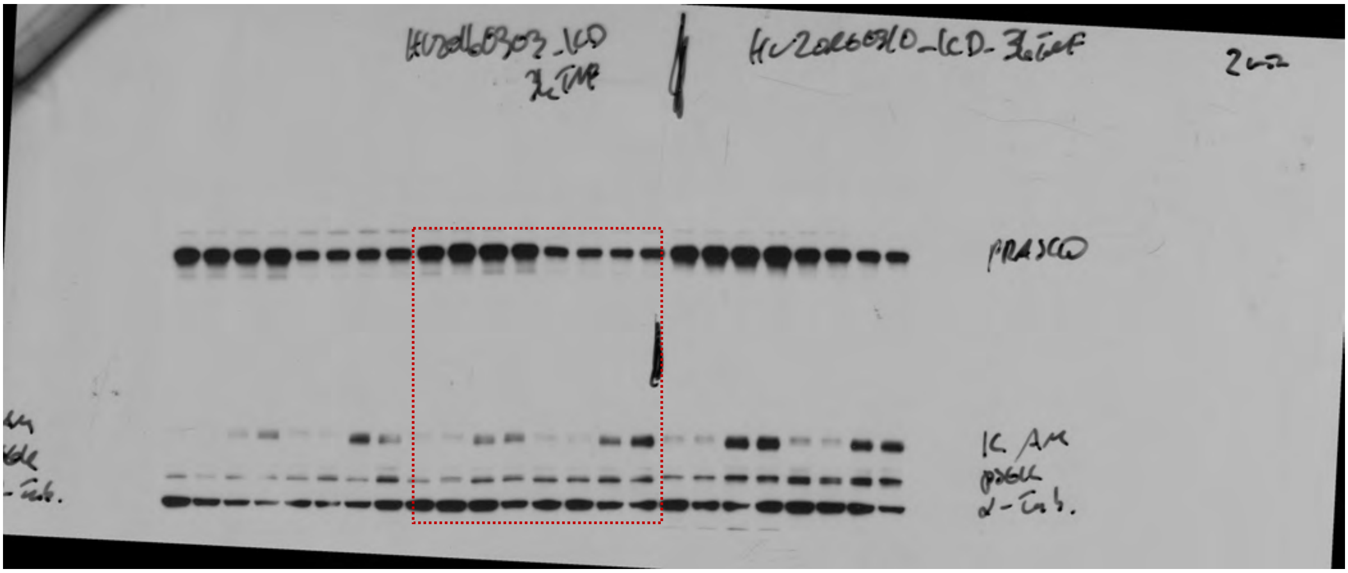

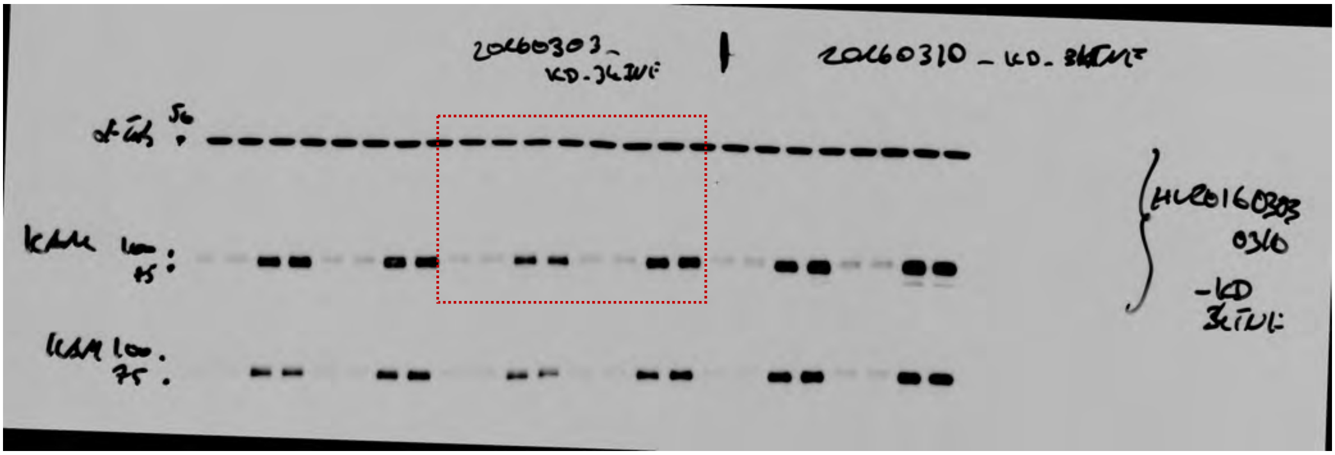

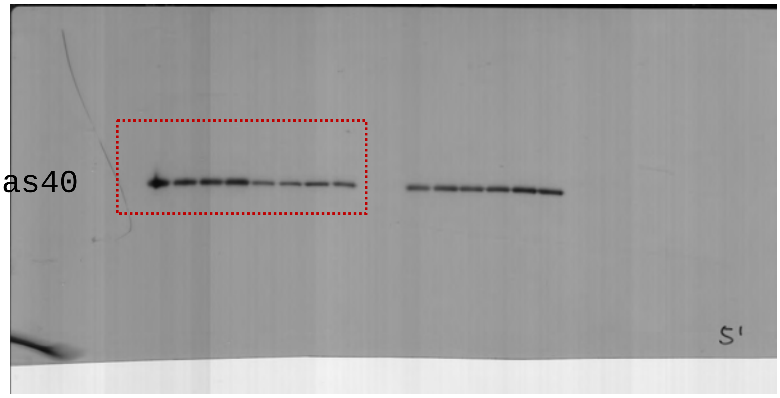

p-pras40

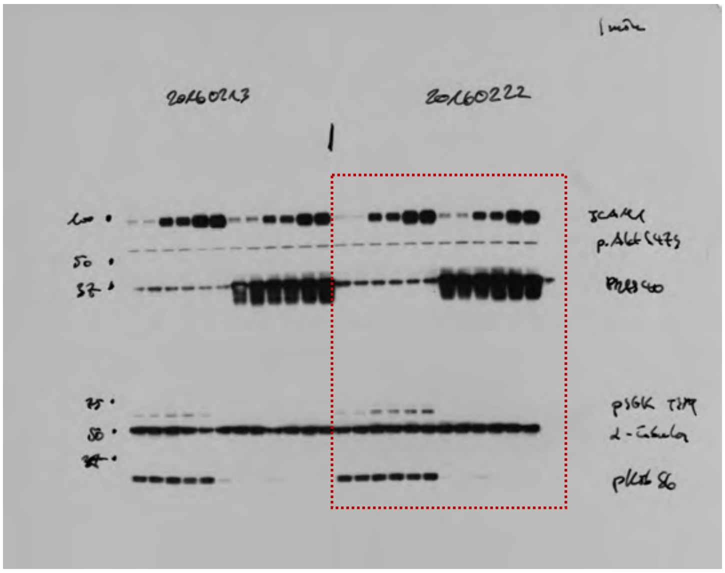

ICAM
Short exposure

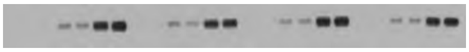

Supplementary Figure S7. Raw data blots figure 3

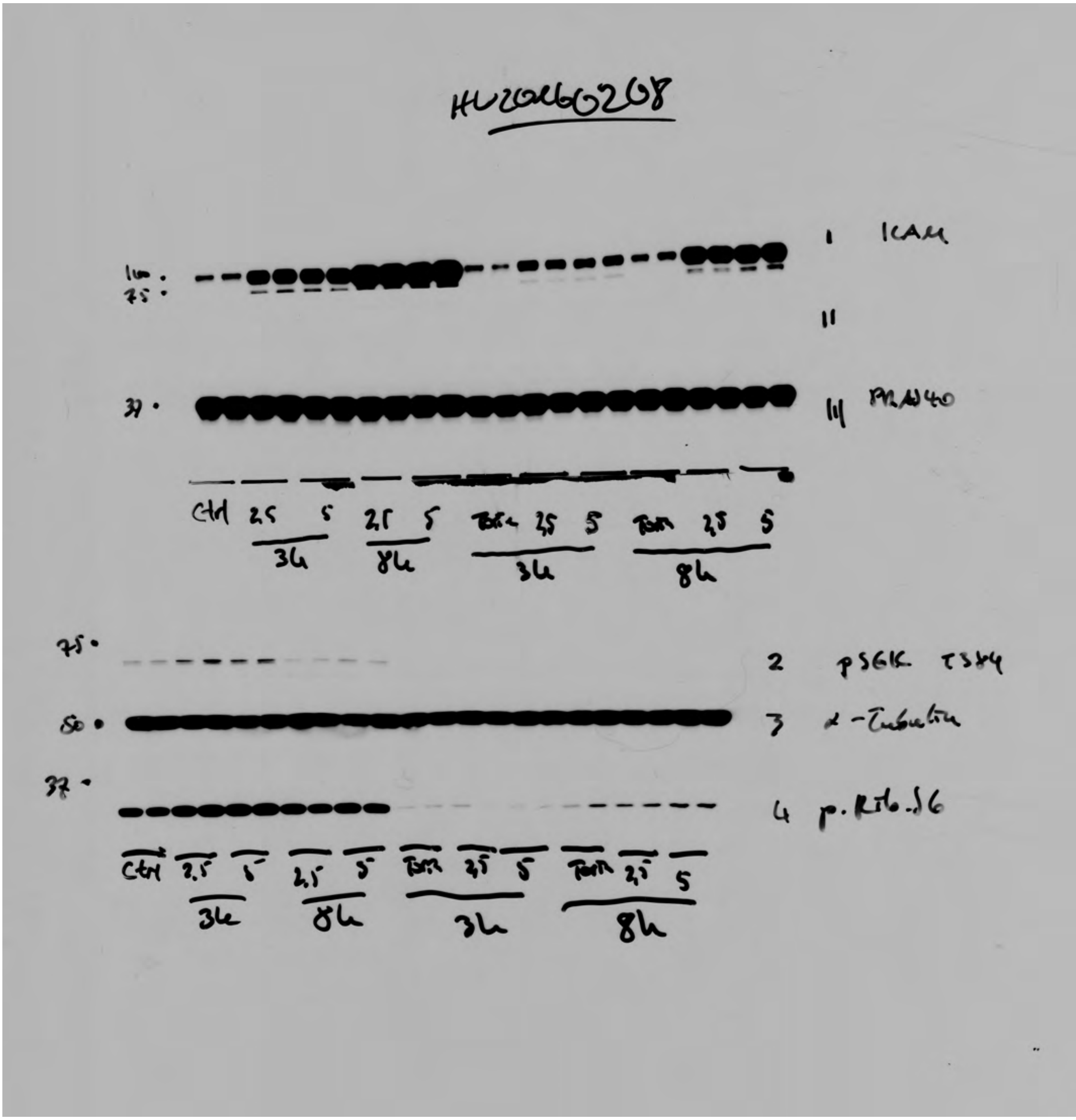

ICAM Low exposure

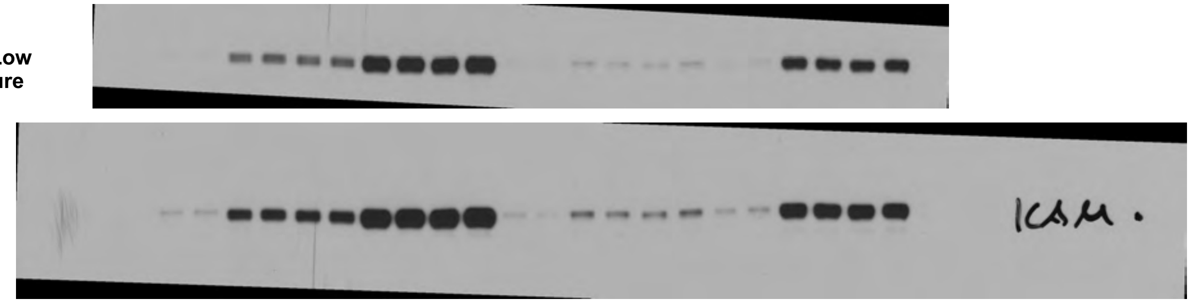

Supplementary Figure S8. Raw data blots figure 5
